# The experiences of newly qualified nurses in intensive care unit: a qualitative meta-synthesis

**DOI:** 10.3389/fmed.2024.1458845

**Published:** 2024-10-30

**Authors:** Ligang Wang, Yuan Chen, Haiyan Yu, Linjing Wu, Aijun You, Xutong Zheng, Yunfei Zhang

**Affiliations:** ^1^Xiamen Cardiovascular Hospital, Xiamen University, Xiamen, China; ^2^Xiamen Hospital, Zhongshan Hospital, Fudan University, Xiamen, China; ^3^Department of Public Service, The First Affiliated Hospital of China Medical University, Shenyang, China

**Keywords:** meta-synthesis, newly qualified nurses, intensive care unit, experience, qualitative research

## Abstract

**Background:**

Clinical rotation practicum provides NQNs with more opportunities to learn their professional knowledge and develop basic nursing skills. ICU is often used as one of the clinical practicum departments for NQNs. Nursing shortages have been particularly felt in ICU. Due to the characteristic fast-paced working environments, high acuity of patient care, and technical complexities of an ICU. The experience of NQNs is highly stressful and challenging in these settings, which hinders their professional development and impacts patient care.

**Aims:**

The study aimed to systematically review and synthesis the findings of qualitative studies exploring experience of NQNs in ICU rotation, to provide a basis for enhancing the quality of clinical nursing.

**Methods:**

Nine databases were systematically searched for relevant publications from inception until February 2024. All qualitative studies in English and Chinese that explored the experience of NQNs in ICU rotation were collected. Two independent reviewers selected the studies and used The Joanna Briggs Institute Critical Appraisal Tool to evaluate the quality of the studies. Meta-synthesis was performed to integrate the results.

**Results:**

A total of 13 studies revealed five descriptive themes and 14 sub-themes: ICU readiness, physical and psychological stress, positive self-perception, developing relationships, and ICU reflections.

**Conclusion:**

Standardized training in ICU, the working ability of NQNs is exercised and their professional quality is improved. However, it is also adversely affected by various stressors. The appropriate guidance and monitoring should be given by hospital managers, so as to promote the development of NQNs and enhance the quality of clinical nursing.

**Systematic review registration:**

https://www.crd.york.ac.uk/PROSPERO/, CRD42023475257.

## 1 Introduction

According to the World Health Organization (WHO) predictions based on current trends, the worldwide nursing workforce will experience shortages of 5.7 million by 2030 (1). Newly qualified nurses (NQNs) leaving their positions shortly after they start work for various reasons is a significant factor contributing to the shortage of nurses. With almost 30% of NQNs leaving their position during their first year of employment, the rate is much higher than the more experienced nurses (2).

The Institute of Medicine (3) published a seminal report, “The Future of Nursing: Leading Change, Advancing Health” in the early 21st century. The report advocated for NQNs to undergo Nurse Residency Programs (NRPs) to become nurses to establish a professional identity and improve the quality of nursing. Since then, NRPs have received garnered significant attention. The United States, Australia, Scotland, Japan, and China have relevant regulations for the NRPs. Although the training cycles vary greatly, they all propose to carry out NRPs in the Intensive Care Unit (ICU). For example, in the United States, NRPs last for 12 months and are primarily conducted in a designated department ICU and general ward (4). China’s Ministry of Health (5) has issued regulations requiring NQNs in hospitals to undergo 2 years of training in four departments, internal medicine, surgery, ICU, and emergency. The program, mode, and cycle of NRPs vary from place to place due to different cultures and medical conditions, NRPs in ICU is widely regarded as essential and useful for nurses’ career, which helps NQNs to master the necessary work skills and quickly adapt to clinical nursing work.

The ICU is where acute and critical patients are gathered in the hospital. The technical complexities require nurses to have strong nursing skills, meanwhile, it also brings intense and heavy work intensity to nurses. Therefore, the NQNs are vulnerable to experience significant emotional exhaustion, stress, and burnout (6). NQNS refers to standardized training for nurses within 1 year of employment, who are in the critical transition from students to clinical frontline professional practitioners, and they will encounter the significant impact of ‘role change’ (7). NQNs rotate into ICU where they face fast-paced patient situations, including emergency response situations, they not only lack experience, but also knowledge, confidence, entry-level clinical judgment ability, and cooperation in the resuscitation of critically ill patients. Without a doubt, this puts forward higher requirements on NQNs’ learning. As newcomers to the workplace, and they are faced with numerous new demands and challenges, NQNs’ experience of early practice represents a significant stage of building confidence and professional identity (8), the practical development of NQNs during ICU can have a significant impact on their future work. Meanwhile, cultivating the knowledge and skills required by NQNs to care for and manage critically ill patients is highly important to increase the ICU specialty nurse reserve.

In recent years, scholars have increasingly focused on the experiences of NONs during their rotation to the ICU. As a result, there has been a gradual increase in related qualitative studies. However, a single qualitative study may not fully capture the breadth of NONs’ experiences during their rotation in the ICU. Therefore, the purpose of the present study is to synthesize the literatures on the experiences of NONs from rotating into ICU. One of the main objectives was to establish an evidence-based foundation for designing targeted interventions that address the actual needs of NONs and improve training models.

## 2 Methods

### 2.1 Design

The purpose of this review is to identify, evaluate, and integrate data from qualitative researches that describe NQNs’ experience in ICU rotation. This qualitative meta-synthesis was conducted following JBI methodology for systematic reviews of qualitative evidence. It was registered in PROSPERO (CRD42023475257) and reported according to the Preferred Reporting Items for Systematic Reviews and Meta-Analyses (PRISMA) guidelines (9).

### 2.2 Search strategy

**Figure 1:**
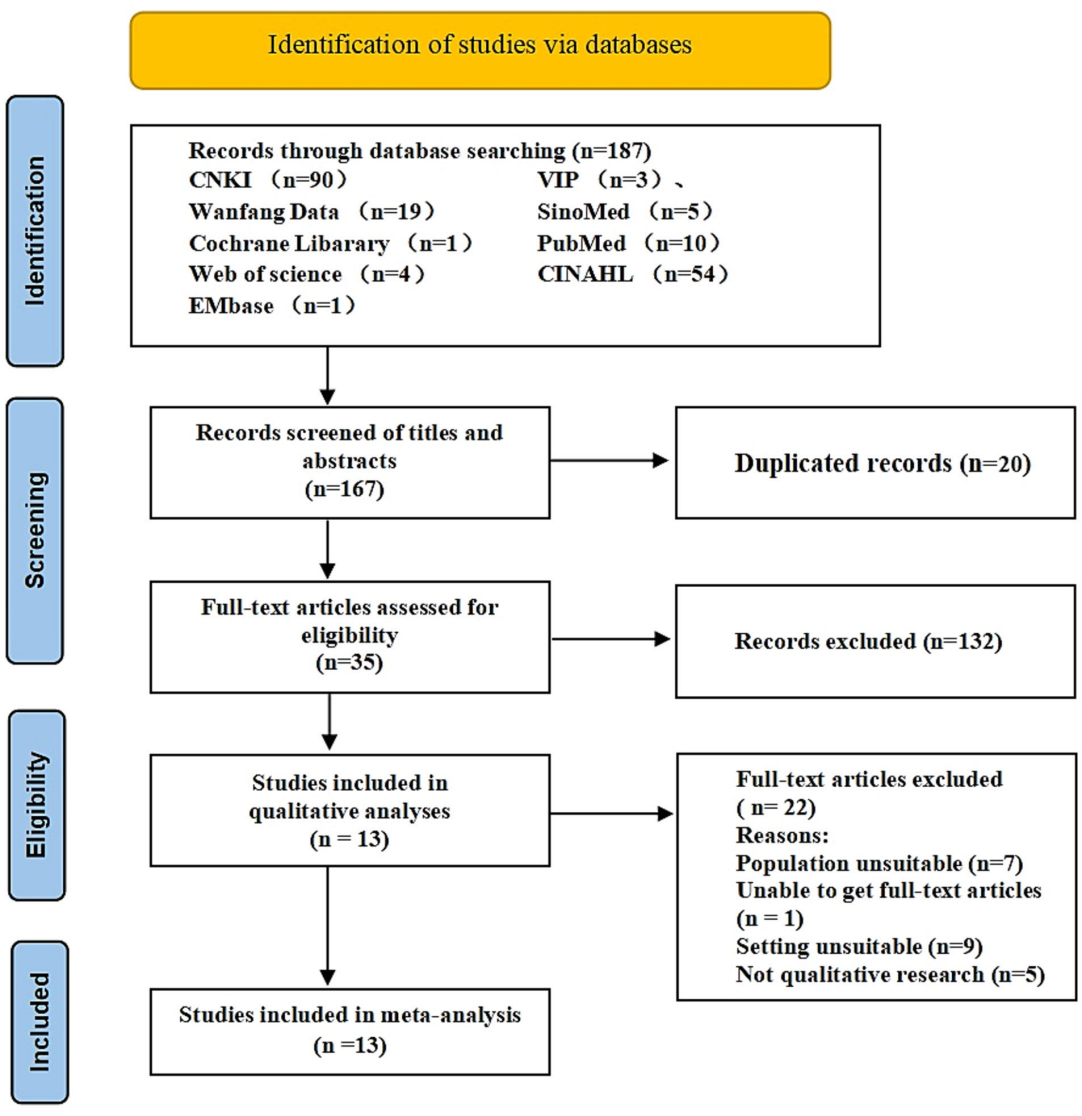
PRISMA flow diagram of the literature search and selection procedure for inclusion of qualitative studies.

Using JBI three-step retrieval strategy for literature retrieval. Step 1: Conduct a preliminary search on PubMed and CINAHL (EBSCO) databases. Analyze the vocabulary in the title and abstract, as well as the topic words of the article. Step 2: use a comprehensively search of English and Chinese databases, including PubMed, Web of Science, The Cochrane Library, CINAHL (EBSCO), Embase, China National Knowledge Infrastructure, WanFang Data Knowledge Service Platform, VIP Database, and China Biology Medicine. The search was conducted by two researchers (WLG and YHY) using a computer from the inception of these databases to February 2024, utilizing a combination of theme words. Step 3: Trace the references included in the literature and supplement the relevant literatures. We made different retrieval strategies according to different databases, the PubMed searching strategy was displayed in Table 1, and the search strategies of other databases were displayed in Supplementary material. The results of the search are presented in the PRISMA flow diagram presented as Figure 1.

**Table 1:** Searching strategy in PubMed.

PICoS	Step	Search terms
Types of participants (*P*)	#1	((((newly graduated nurses [Title/Abstract]) OR (newly qualified nurses [Title/Abstract])) OR (newly employed nurses [Title/Abstract])) OR (newly registered nurses [Title/Abstract])) OR (newly licensed nurses [Title/Abstract])
Types of phenomena of interest (*I*)	#2	(Intensive Care Units [MeSHTerms]) OR (((Intensive Care Units [Title/Abstract]) OR (Intensive Care Unit [Title/Abstract])) OR (Unit,Intensive Care[Title/Abstract]))OR(ICU Intensive Care Units[Title/Abstract])
	#3	(Train[Title/Abstract])OR(standardized training[Title/Abstract]))OR(pre-servicetraining[Title/Abstract])
Types of contexts (*Co*)	#4	(experience[Title/Abstract])OR(feel[Title/Abstract])
Types of studies (*S*)	#5	(qualitative research[Title/Abstract])OR(qualitative method[Title/Abstract]))OR(qualitative study[Title/Abstract]))OR(phenomenolog[Title/Abstract]))OR(lived experience[Title/Abstract]))OR(hermeneutic[Title/Abstract]))OR(Heideggerian[Title/Abstract]))OR(husserl[Title/Abstract]))OR(groundedtheory[Title/Abstract]))OR(ethnograph[Title/Abstract]))OR(casestudy[Title/Abstract]))OR(discourseanaly[Title/Abstract]))OR(interview[Title/Abstract]))OR(actionresearch[Title/Abstract]))OR(Olparticipantobserv[Title/Abstract]))OR(fieldnote[Title/Abstract]))OR(focusgroup[Title/Abstract]))OR(Colaizzi[Title/Abstract]))OR(contentanaly*[Title/Abstract]))OR(thematicanaly*[Title/Abstract]))OR(Giorgi[Title/Abstract]))OR(Manen[Title/Abstract]))OR(constant comparison[Title/Abstract]))OR(constant comparativea nalysis[Title/Abstract])
	#6	#1 AND #2 AND #3 AND #4 AND #5

### 2.3 Study selection

The search results were imported into EndNote X9 software. After duplicated studies being removed, two researchers (WLG and YHY) independently screened the titles and abstracts, removing studies that did not meet the criteria, which are shown in Table 2. Then, the full texts were evaluated and studies meeting the criteria were included. Two researchers verified the included studies. Any disagreements were resolved through discussion with the third researcher (CY).

**Table 2:** Inclusion and exclusion criteria.

Inclusion criteria	Excluded criteria
Participant (P): NQNs who were starting clinical work in ICU.	They were reviews, conference abstracts, quantitative studies, commentaries, and research protocols.
Interest of phenomenon (I): the real experience or feelings generated by NQNs during ICU rotations.	Qualitative data could not be extracted in a mixed method study.
Context (Co): NQNs undergo a phase of standardized clinical training in the all ICUs-including specialist ICU’s (e.g., medical, surgical, cardiac, neurological, and burns).	Unable to obtain full-text, repeatedly published or incomplete literature.
Study design (S): qualitative research, or mixed methods research (extracting only the qualitative component), includes phenomenology, grounded theory, action research, and ethnography.	Any other language.
Language is limited to English and Chinese.	

### 2.4 Data extraction and synthesis

Data extracted from the selected studies included standardized information, such as authors, sample size, qualitative methods employed, research aim and themes. The format was predetermined by the authors (see Table 3).

**Table 3:** Characteristics of included studies.

Author, year	Country	Participants	Method	Aim	Themes
Li M. et al. 2021 (11)	China	10 NQNs in ICU	Descriptive phenomenology Semi-structured in-depth interviews.	The real inner experience of NQNs in ICU.	Five themes were identified: (1) Physiological stress; (2) Conflict between practical needs and individual capacities; (3) Complex interpersonal relationships (4) Organizational Factors Sense of Belonging; (5) Career development in the context of the epidemic.
Liu W. N. et al. 2020 (16)	China	12 new employed nurses rotate in the cancer ICU	Phenomenological research semi-structured interview method.	To explore and analyze the psychological states and real experience of new employed nurses in ICU.	Four themes were identified: (1) Unfamiliarity with the work environment and instrumentation; (2) Inner confusion and fear (burden of thought); (3) Lack of effective communication; (4) Different experiences of guilt and achievement.
Xiang M. M. et al. 2023 (15)	China	13 NQNs in ICU	Interpretive Phenomenology Staged In-depth Interviews.	Understanding the experience of new nurses in ICU.	Three themes were identified: (1) Period of self-denial: I am an inexperienced performer; (2) Striving for advancement period: I am struggling to face the challenges of work; (3) Acceptance period: I am getting used to my new role.
Zhang J. N. et al. 2021 (14)	China	12 nurses trained in Surgical Intensive Care Unit (SICU), Cardiovascular Intensive Care Unit (CICU), Neurosurgical Intensive Care Unit (NICU)	Phenomenological research semi-structured interview method.	Understanding the experience of new nurses in ICU.	Three themes were identified: (1) Inadequate job competence and lack of encouragement; (2) Difficulty in team integration; (3) Poor welfare benefits and insufficient reflection of career value.
Zhang XZ et al. 2017 (17)	China	12 ICU standardized training new nurses	Phenomenological research Semi-structured interview method.	Understanding psychological experience of unit belonging among new nurses in ICU.	Three themes were identified: (1) Lack of job competence, lack of encouragement; (2) Difficulty in team integration; (3) Poor welfare benefits, insufficient reflection of career value.
O’Kane et al. 2012 (31)	England	Eight NQNs in ICU	A comparative, qualitative approach.	Investigate different experiences of newly qualified nurses starting their career in critical care.	Four themes were identified: (1) Expectations; (2) Challenge; (3) Preconceptions; (4) Support.
Della Ratta et al. 2016 (32)	The United States	Eight novice nurses in ICU and Emergency department (ED)	Qualitative interpretive phenomenological analysis.	To explore graduate nurses’ experiences of caring for deteriorating patients during the first year of practice.	Three themes were identified: (1) Dwelling with uncertainty; (2) Building me up; (3) A new lifeline: Salient being.
Saghafi et al. 2012 (33)	Australia	10 new graduate nurses in ICU	Phenomenological approach	The experiences of new graduate nurses in ICU.	Three themes were identified: (1) Interaction with patients; (2) Interaction with other members of ICU team; (3) Who is approachable?
McKenzie et al. 2021 (12)	Australia	Eight newly qualified registered graduate nurses in NICU	Narrative inquiry approach	To explore the experiences of newly qualified registered graduate nurses’ clinical and professional learning experiences.	Four themes were identified: (1) Feeling unprepared; (2) Horizontal violence; (3) Supportive structural environment; (4) Seeking feedback.
Davenport et al. 2000 (18)	The United States	Eight new nurses trained in ICU	Interpretive phenomenological study	Understanding the experience of beginning a professional life as a new ICU nurse.	Ten themes were identified: (1) Finding a Home; (2) These are a Few of the Hardest Things; (3) Family Care; (4) Relationships with Colleagues: Roommates and Neighbors; (5) Socialization; (6) The Power of Team Work; (7) Asking Questions; (8) Emergencies and Deaths: Helping with an Arrest, Learning the Skills, and Feeling the Emotions; (9) Watchfulness; (10) Moving on: No Longer the New Kid on the Block.
Lewis-pierre et al. 2013 (13)	The United States	Seven new graduates working in ICU	The qualitative grounded-theory study	Explain workplace readiness and needs of new graduates entering the intensive care unit (ICU).	Four themes were identified: (1) Embracing the new ICU role; (2) Overwhelming experience of performance ambiguity or anxiety; (3) Adapting to the ICU; (4) Embodying the new ICU RN role.
Wiles et al. 2010 (19)	The United States	Six newly graduated nurses in the critical care	Phenomenological methodology	To explore the experiences of NQNs beginning their career in ICU.	Three themes were identified: (1) Developing confidence in practice; (2) Seeking assistance; (3) Decision making.
Hussein et al. 2017 (34)	Australia	72 new graduate nurses in ICU	A convergent mixed methods design.	Examine change in new graduate nurses’ perceptions over the 12-month Transitional Support Program.	Two themes were identified: (1) Orientation and Transitional Support Program as foundation for success; (2) Developing clinical competence.

### 2.5 Appraisal of methodological quality

Studies considered for inclusion in the review were critically appraised with the Joanna Briggs Institute (JBI) 10-item standardized critical appraisal checklist for qualitative studies by two independent reviewers (see Table 4 for appraisal results). As reported in a previous meta-synthesis (10), a minimum of “yes” for six domains was required for inclusion. Literature that meets all criteria has a low likelihood of bias and is graded A. Literature that meets some criteria has a medium likelihood of bias and is graded B. Literature that does not meet the criteria at all has a high likelihood of bias and is graded C. The final inclusion level of B is based on the results of a survey of the literature. If there is any difference in the evaluation results, the third researcher will resolve the conflict. The final inclusion of the literature above level B.

**Table 4:** Critical appraisal results for included qualitative studies using the JBI-qualitative critical appraisal checklist.

References*	Q1**	2	3	4	5	6	7	8	9	10	quality level
Li M. et al.	Y	Y	Y	Y	Y	N	N	Y	Y	Y	B
Liu W. N. et al.	Y	Y	Y	Y	Y	N	N	Y	N	Y	B
Xiang M. M. et al.	Y	Y	Y	Y	Y	N	N	Y	Y	Y	B
Zhang J. N. et al.	Y	Y	Y	Y	Y	N	N	Y	N	Y	B
Zhang X. Z. et al.	Y	Y	Y	Y	Y	N	N	Y	N	Y	B
O’Kane et al.	U	Y	Y	Y	Y	Y	Y	Y	Y	Y	B
Della Ratta et al.	Y	Y	Y	Y	Y	Y	Y	Y	Y	Y	A
Saghafi et al.	Y	Y	Y	Y	Y	Y	Y	Y	Y	Y	A
McKenzie et al.	Y	Y	Y	Y	Y	N	N	Y	Y	Y	B
Davenport et al.	Y	Y	Y	Y	Y	Y	Y	Y	Y	Y	A
Lewis-pierre et al.	Y	Y	Y	Y	Y	Y	Y	Y	Y	Y	A
Wiles et al.	Y	Y	Y	Y	Y	Y	Y	Y	Y	Y	A
Hussein et al.	U	Y	Y	Y	Y	N	N	Y	Y	Y	B

*Critical appraisal (*n* = 10) of (Y = yes; N = no; U = unclear; NA = not applicable). **Question = Q. Domains: (1) Congruity between the stated philosophical perspective and the research methodology; (2) Congruity between the research methodology and the research question or objectives; (3) Congruity between the research methodology and the methods used to collect data; (4) Congruity between the research methodology and the representation and analysis of data; (5) There is congruence between the research methodology and the interpretation of results; (6) Locating the researcher culturally or theoretically; (7) Influence of the researcher on the research, and *vice-versa*, is addressed; (8) Representation of participants and their voices; (9) Ethical approval by an appropriate body; (10) Relationship of conclusions to analysis, or interpretation of the data.

## 3 Results

### 3.1 Search outcomes

The initial search returned 187 articles, and 167 studies were included after removing duplicate articles and reading the title and abstract. After reading the full-texts, 13 studies were finally included. (Figure 1).

### 3.2 Study information and the methodological quality of the included literatures

In the present review, 13 studies were included with a total of 124 NQNs. The studies were conducted in China (*n* = 5), the United States (*n* = 4), Australia (*n* = 3), and England (*n* = 1), characteristics of included studies are shown in Table 3. Of the 13 studies included, five were of grade A quality and the rest of them were grade B quality.

### 3.3 Qualitative meta-synthesis

**Figure 2:**
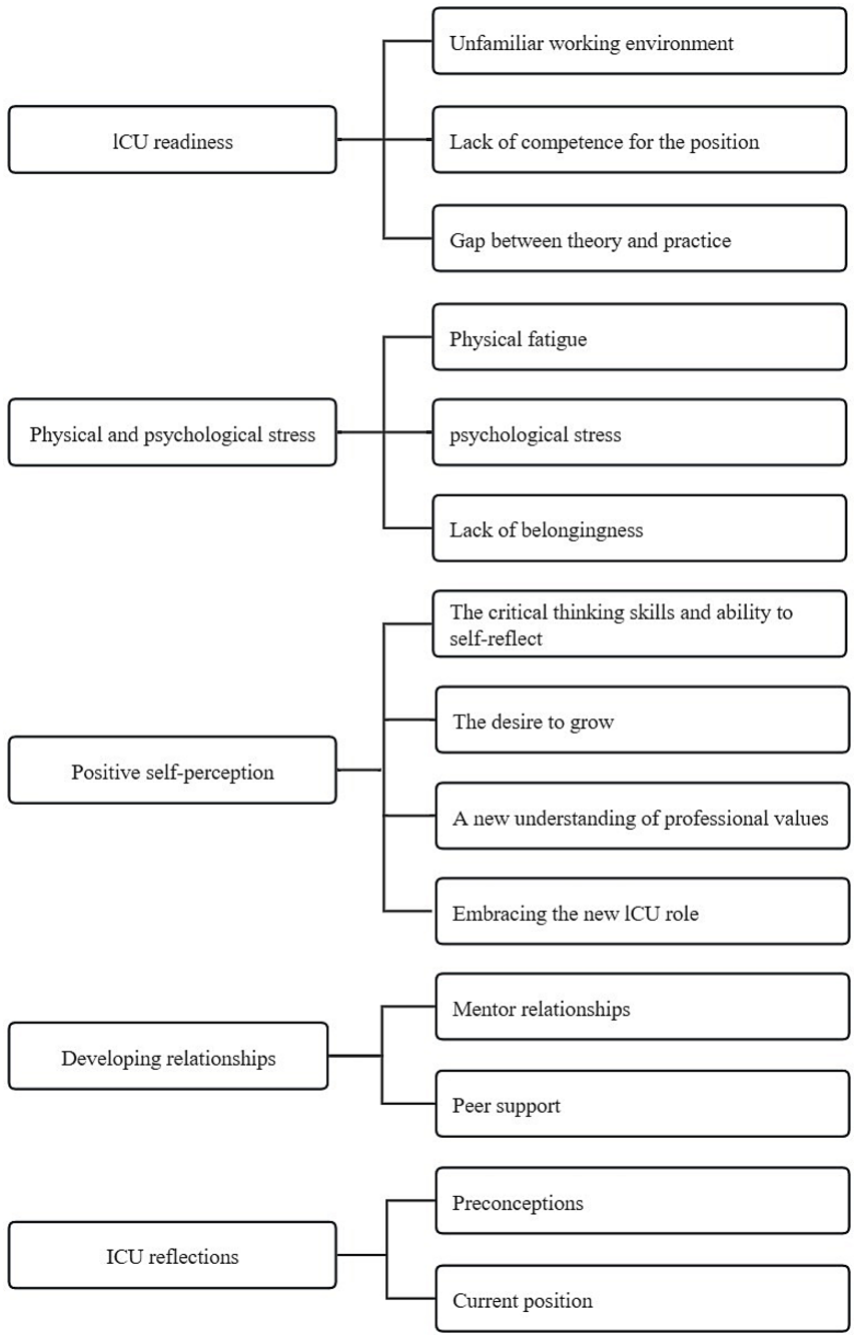
Thematic synthesis findings.

This study synthesized the results of a qualitative study of 124 NQNs, generating five themes (ICU readiness, physical and psychological stress, positive self-perception, developing relationships and ICU reflections) and 14 sub-themes. A summary of the themes is shown in Figure 2.

#### 3.3.1 ICU readiness

##### 3.3.1.1 Unfamiliar working environment

The ICU which differs from ordinary wards, NQNs feel unfamiliar due to the presence of advanced instruments, equipment, and medical materials. *“It’s not like a normal ward, it’s all transparent single rooms, and a lot of the instruments and equipment are different, so I find it very refreshing, but I just do not know how to use it”* (11). *“I was really excited when I found out I was coming here. But at the same time, I was a bit nervous, knowing that I have not done any neonates whatsoever”* (12).

##### 3.3.1.2 Lack of competence for the position

Intensive Care Unit patients are characterized by acute and critical illnesses, necessitating nurses with solid professional knowledge base and excellent clinical skills. *“I learned medications in the nursing schools but the discussions did not include all the ICU medications such as beta-blockers and analgesics”* (13). *“I could not draw blood gas analysis at first. I had to ask the teacher for help, and many times I had to ask the teacher to draw it. I was so depressed that I lost confidence in myself, and it took me a long time before I could draw arterial blood on my own”* (14). *“I was really excited when I found out I was coming here. But at the same time, I was a bit nervous, knowing that I have not done any neonates whatsoever”* (12).

##### 3.3.1.3 Gap between theory and practice

There is a gap between theoretical knowledge and practical teaching in school and clinical reality. Many situations involving ICU patients cannot be predicted, and the knowledge acquired in the past may not be sufficient to cope with the real operation. *“I think the content of the training is too theoretical, I cannot even understand it well after the lecture, and I cannot remember what was said, I do not have any concept. I think the teachers ought to take us to the clinical practice, so that we can combine theory and practice, otherwise we do not know what to do”* (14). *“The biggest feeling is that I feel like a blank sheet of paper, the kind that drags my colleagues down, and I feel like I cannot do anything right. I feel like I cannot do anything right. I should have learnt everything in the ICU all over again”* (15).

#### 3.3.2 Physical and psychological stress

##### 3.3.2.1 Physical fatigue

The high intensity of work in the ICU and the unique nature of the shift system makes many ICU rotating NQNs feel physically fatigued. *“Sometimes 3 night shifts a week, it’s hard, and the physiological period is also a bit disturbed, and a sleep, not very regular anymore”* (11). The diseases of ICU patients are complex and change rapidly is overwhelming, confusing, and unfamiliar to the NQNs. *“It’s too busy here, I’m simply too busy on my own, I have to take care of serious patients, I have to admit new patients and post-operative patients, and I also have to transfer patients, my legs are so sore that I cannot wait to crawl back home after my shift, or I can just lie down in the department for a while and then go home again”* (14).

##### 3.3.2.2 Psychological stress

Newly qualified nurses rotating into the ICU are confronted with changes in the condition of critically ill patients, co-operating with the resuscitation process, and being unfamiliar with emergency instruments. These situations can evoke vivid imagery and lead to distress and psychological stress. *“I do not know why, but when I walked into this department, I had an inexplicable sense of fear. Feeling very eerie, see the patient lying there motionless and then hear a* var*iety of alarms, I was numb, I think I am not suitable for ICU work”* (14). *“The working condition is very tense, I do not dare to leave the patient at will for fear that he might be in danger”* (16).

##### 3.3.2.3 Lack of belongingness

A sense of being accepted and recognized is considered as an important factor in the process of learning. The lack of belongingness is a major challenge faced by NQNs during their practicum in the ICU. A portion of the NQNs felt alienation and loneliness in the ICU, possible due to the indifference of nurses and the environment of the ICU. *“I do not feel safe working here either, I do not feel like it’s my department, I just feel like it’s a temporary place to work, and I’m just numb to doing things every day, and I’m not happy at all”* (14). *“I love to learn, but I do not get to go out on study trips or attend nursing conferences. I feel like I’m floating, like me do not matter, like nothing belongs to me, like I’m insecure, like no one remembers me, like you do not even have a name”* (17). *“It does not feel like we are really being groomed to be in ICU, just let us do the operations for them”* (11).

#### 3.3.3 Positive self-perception

##### 3.3.3.1 The critical thinking skills and ability to self-reflect

This theme encompassed the NQNs’ development of critical thinking skills and ability to self-reflect. Critical thinking is challenging for the new ICU nurse to develop. Improvement in NQNs’ competencies was an essential theme of the studies. Competencies were described as the complex NQNs traits that entailed knowledge, skills, and attitudes. *“I was so busy and so overwhelmed that night. It was such a pain to have an isolation patient. I felt like my other two patients did not get my attention. When she died, I felt like I could have done something different … I could have been a little bit more compassionate. I beat myself up for that”* (18).

##### 3.3.3.2 The desire to grow

Due to numerous differences emerging between ICU and non-ICU clinical practicum, the participants realized that they lacked the appropriate specialized knowledge to implement their practicum skills, but indicated their desire to have the knowledge. Meanwhile, NQNs were able to identify their professional deficiencies, enhance their skills through diverse training practices, and set clearer goals for their growth and development during ICU rotations. *“Now I will actively strive for hands-on opportunities, I remember once, an arterial puncture placement opportunity I did not seize because I was too panicked, now I think a little regret”* (15).

##### 3.3.3.3 A new understanding of professional values

Positive professional values among nurses contribute to the development of appropriate professional attitudes, which in turn enhance the quality of clinical care and nursing satisfaction. *“I used to want my work to be work and my life to be life, but after I got into nursing I realized that I needed to spend more time filling my job … Keep up the good work and get through it to be a better version of yourself”* (15).

##### 3.3.3.4 Embracing the new ICU role

Some NQNs said that as the practicum progressed, they adapted to the ICU environment. *“After work, I will check one by one if there is anything I have missed, tidy up the patient’s bed unit and remove anything that cannot be used, so that I can feel more at ease when I hand over the patient to someone else”* (15). As NQNs became more adaptable, they became more confident of their performance in the ICU. *“It was easy enough. I’m not cocky. I am good-I know that”* (19).

#### 3.3.4 Developing relationships

Developing relationships with patients, families, mentor and peers was an important aspect of the NQNs’ rotation in ICU.

##### 3.3.4.1 Communication with patients

Newly qualified nurses found it difficult to communicate with critically ill patients, due to barriers such as sedation or delirium. They feel satisfied when they overcome these barriers to building a relationship with the patients. *“I held his hand and I talked to him by his first name. He’s confused at times and I’m trying to make sure he knows where he is and who he is because he does not always know that … The patient’s comfort, that he sleeps good when I’m there, is important and I’m proud of that”* (18).

##### 3.3.4.2 Mentor relationships

Newly qualified nurses overwhelmingly identified the negative impact of having multiple mentors. Lack of consistency meant that they were having to prove themselves to a new person each day, different mentors underestimating or overestimating their abilities. *“I felt like I had a different preceptor [mentor] everyday … They never asked me what I felt comfortable doing. I had such a conflict about techniques. One would say you should really do it this way. Another would want me to do the same thing another way. I was so overwhelmed”* (18). *“My preceptor is the last person I will go to. I just do not feel that I can talk to her”* (19).

##### 3.3.4.3 Peer support

The NQNs were keen to have their peers’ support, which confirmed the importance of the instructors’ encouragement in building their confidence during the practicum. The positive attitude of peers had a positive impact on the overall learning process. *“There were a lot of other GNPs [graduate nurse program] starting with me or around about the same time as me. you have people to relate to, you can understand”* (13). *“Once a doctor wanted to do fibreoptic bronchoscopy on a patient, the teacher asked me to co-operate with this doctor, and I carried out the operation according to his request, after he knew that I was a new colleague, this doctor complimented me to the teacher, I was especially happy that day”* (15). *“Honestly, I think that the key to like being comfortable when you’re a new grad is having strong people that you work with and people that are willing to help you”* (19).

#### 3.3.5 ICU reflections

Newly qualified nurses in this review who had done their practicum in the ICU realized that nurses played a key role in the ICU, which provided optimal, quality healthcare to ICU patients. NQNs were motivated to work in ICU by a “dream,” “calling” or get their “foot in the door” by gaining experience as a Healthcare Assistant. ICU was an appropriate place to start their career, most NQNs felt that it was dependent on previous experience, but others did not think this was necessary. *“For someone with absolutely no past nursing experience who from undergraduate go out and do GNP [graduate nurse program] placement, ICU should not be the first placement. I think you should integrate all your basic nursing skills first…”* (18). As they became aware of the value of professionalism, the NQNs also chose to become critical care nurses in the future. *“I felt that I could do a better job here (ICU) than on an understaffed ward. I feel that on a ward, if there is not enough staff, I could not give the standard of care which is not only acceptable to the patient but to me and the reason I went into nursing”* (13).

## 4 Discussion

NONs face a lot of challenges in the ICU rotation due to factors such as the unique environment, lack of theoretical knowledge and inadequate practical skills. NONs’ own professional quality has been enhanced through learning and practice, which is crucial for their personal career development and nursing teams. This study summarized the existing evidence on the experiences of NONs during their rotation in the ICU across 13 studies.

“*ICU Readiness*” encompasses how prepared NQNs felt to work in ICU, including unfamiliar working environment, lack of competence for the position, and the gap between theory and practice. The working environment in the ICU differs from the ward, NQNs are unfamiliar with the equipments and work content in the ICU, which makes them feel uncertain and unable to start to work. Mentors should assist NQNs in acclimating to the ICU environment and training them in equipment usage. Hospital managers could invite senior NQNs to participate in a symposium aimed at sharing and learning experiences to help them rotate into the ICU working environment and complete their role conversion as soon as possible. Mentors can establish the NQNs Facebook or WeChat Messenger groups for NQNs to share course materials, interact online, and offer tailored guidance and support. Multiple studies indicate that NQNs lack competence for the position during the ICU rotation and are not recognized by senior nurses and doctors (12, 15, 18). It cannot be fully integrated into the department; therefore, it is urgent to explore training methods to improve competence for the position. NQNs’ competence for the position including strong personal characteristics, critical nursing ability, interpersonal communication skills, clinical thinking ability, and professional development capabilities. Enhancing NQNs’ competence for the position can assist them in adapting to nursing work and becoming proficient ICU nurses as quickly as possible. For hospital managers, strategies such as the clinical path teaching method, scenario simulation teaching method and Potter-Laul’s comprehensive incentive model can enhance the competence of NQNs in their roles. The “one-on-one” teaching mode can further deepen NQNs’ understanding of ICU knowledge. Regardless of the method used to enhance NQNs’ comprehension in their positions, the ultimate objective is to boost their self-confidence and enthusiasm for their careers, and to deliver high-quality nursing services to patients. It is essential for them to strive for continuous improvement in comprehensive quality, engage in more practice, update professional knowledge, learn new technologies, establish reasonable goals, and serve as the main force of the ICU nursing team (20).

Multiple studies (14, 15) have shown poor integration of theory and clinical practice in the standardized training content for NQNs. The ICU working environment is unique and involves higher nursing risks compared to the general ward. The department will provide theoretical training as part of the pre-job training for new nurses before NQNs enter the ICU. However, NQNs still feel that the training content needs additional supplementation. The results of the study (21) that NQNs have a high degree of recognition of first aid skills training and an urgent need for training in comprehensive first aid theoretical knowledge, individual nursing first aid operational skills, and overall first aid competence. Moreover, the level of NQNs in terms of operational skills is poor, and the percentage of nurses who can proficiently master the relevant first aid skills is low. Therefore, to cultivate the all-round development of NQNs, nursing managers should not simply follow the training plan of the department to train the nurses in clinical teaching. They should also conduct individualized training according to the NQNs’ personality characteristics and learning needs.

Studies have shown that NQNs faces different degrees of physical and psychological stress during the ICU rotation (14, 16). Sleep disorders, headaches, endocrine dysfunction, and other symptoms have also been reported in previous studies to commonly manifest in clinical work, impacting the health of nurses (22). As most of the NQNs are unfamiliar with the work content, they need more time to adapt. At the same time, during their NRPs, they are required to attend more additional theoretical training and exams, which requires more personal time to be invested. The high level of stress and lack of rest are very detrimental to their health. Objective Structured Clinical Examination (OSCE) (23) and Mini-Clinical Evaluation Exercise (Mini-CEX) (24) have been proven to be suitable for evaluating nursing clinical competence in recent years. These efficient models should be considered for the evaluating NQNs. Gillespie (25) speculated that positive coping benefits nurses’ development, which is consistent with our findings. Improving the resilience of NQNs in the ICU in the face of physical and psychological stress is particularly crucial for fostering the physical and psychological health and career development of the NQNs group. Nursing managers could assist NQNs in reducing stress and enhancing resilience levels through group counseling, positive thinking therapy and resilience training (26, 27).

A phenomenological study (28) in Australia noted the different personalities and attitudes among doctors and senior nurses overwhelmed NQNs, and in general, NQNs found communication with doctors relatively uncomfortable. The lack of respect from the experienced nurses toward NQNs. The unstable work environment makes it difficult for most NQNs to integrate into the department team and lack of belonging during ICU rotations. It is often observed that when they encounter problems, they do not actively seek help from their colleagues. The psychological resilience is low, making it easier to react emotionally to problems, leading to increased complaints and dissatisfaction. Studies have shown that the psychological well-being of NQNs is closely related to the social support they receive (13, 18, 19). Support from family, mentors, and colleagues can effectively assist NQNs in enhancing their stress adaptability and psychological adjustment, which is consistent with our findings. Hospital managers can invite clinical educators and physicians to participate in an exchange symposium. Effective communication can help NQNs feel the support of the ICU team, enhancing their sense of belonging. In order to improve the sense of belonging among NQNs and ensure they feel supported by the ICU team, NQNs should also strengthen their professional identity and responsibilities. This can be achieved through meaningful communication with colleagues, expanding their knowledge base, and striking a balance between theoretical understanding and clinical proficiency. These efforts will help foster strong collaborative relationships between senior nurses and NQNs (29).

Good self-knowledge and positive professional identity are favorable factors for nurses’ career development. Correct professional values form the foundation of clinical nurses’ practice, the core of their overall quality, and the prerequisite for enhancing the quality of nursing services (30). Nursing managers should prioritize training NQNs in career planning and ethical conduct to effectively enhance their career prospects and professional ethics. Hospital managers should take the rotation training as an opportunity to actively play the role of excellent nurses’ seniors and clinical nursing experts’ demonstration and leading role, to improve the social status of the nursing profession, to create a positive social atmosphere to make the NQNs realize the value and connotation of the nursing work, and the spirit of the nursing professional values to be applied to the practice, so as to improve the self-efficacy of the NQNs, and to enhance the sense of professional identity and the enthusiasm for work during the ICU rotation. This is highly beneficial for the personal growth and career development of NQNs, and it is crucial for maintaining the stability of the nursing workforce and enhancing the quality of nursing care.

Several studies (13.20.22) have discussed the NQNs’ development of critical thinking skills and the ability of self-reflect. Participants were reflective, describing scenarios they had learned from. They often criticize their own practice, questioning what they could have done better, without appreciating other contributing factors. During their rotations in ICU, NQNs gradually begin to manage patients. When encountering problems, they are no longer primarily frightened and complaining. Instead, they strive to find solutions and learn to confront the challenges encountered during operations. Mentors could modify traditional teaching models, clarify key points and challenging problems, and enhance NQNs’ abilities for self-learning, clinical reflection, and independent thinking through micro-lessons.

## 5 Conclusion

This systematic review by synthesizing 13 qualitative studies on the actual perceptions of NQNs rotation in ICU, which can raise awareness of this population. It is essential to understand and integrate NQNs’ psychological and social changes into management strategies. NQNs face complex patients, heavy work pressure, physical and psychological stress, who often lack of belonging, job competence and interpersonal communication skills during their ICU rotational exposure. Nursing managers ought to develop corresponding policies and provide more learning opportunities to promote NQNs successfully rotate from nursing students to professional nurses and enhance the efficacy of standardized nurse training. This can be achieved by developing relevant policies, offering more learning opportunities, and fostering their professional development, ultimately enhancing the effectiveness of standardized nurse training.

The methodologies of original studies we included are mostly phenomenological studies. It is recommended that future research focus on enhancing the methodological quality. The research on the experience of NQNs during ICU rotations will also require a variety of research methods in the future to explore the factors that influence the experience of NQNs. Establish a reference framework to develop effective intervention measures and enhance training strategies to support the development of NQNs.

## 6 Study limitations

This study has several limitations. Firstly, according to the inclusion criteria, only primary qualitative studies published in indexed journals in English or Chinese were selected. Therefore, gray literature and dissertations were not searched, which might have introduced an information bias. Secondly, the original studies included were from multiple countries, and there are differences in NQNs in different cultures perceive the problem, which may lead to cultural bias in our interpretation as well. Thirdly, the existing literature only provides a phenomenological perspective on the rotation experience of NQNS in the ICU. Additionally, some literature fails to address the influence of researchers’ values and cultural backgrounds on the research results.

## Data Availability

The original contributions presented in the study are included in the article/Supplementary material, further inquiries can be directed to the corresponding author.
