# Supplementary material for: The experiences of newly qualified nurses in intensive care unit: a qualitative meta-synthesis
Source: Front Med (Lausanne). 2024 Oct 30;11:1458845. doi: 10.3389/fmed.2024.1458845 (PMC11557346; doi:10.3389/fmed.2024.1458845)
Supplement: Supplementary file 1 [file Supplementary_material.pdf]

Supplementary Material

1. Search Strategies

（1）Chinese national knowledge infrastructure，CNKI　　88

#1　SU=新护士 OR SU=新入职护士 OR SU=新毕业护士

#2　SU=ICU OR SU=重症监护室 OR SU=重症 OR SU=危重症

#3　SU=规范化培训 OR SU=岗前培训 OR SU=轮转

#4　SU=体验 OR SU=感受 OR SU=体会

#5　SU=质性研究 OR SU=定性研究 OR SU=现象学

#6　#1 AND #2 AND #3 AND #4 AND #5

SU=(新护士 + 新入职护士 + 新毕业护士 + 新执业护士) AND SU=(ICU + 重症监护室 + 重症 + 危重症 + 重症监护 + 监护室) AND SU=(规范化培训 + 岗前培训 + 轮转) AND SU=(体验 + 感受 + 体会 + 体验 + 看法 + 经历 + 想法) AND SU=(质性研究 + 定性研究 + 现象学 + 扎根理论 + 叙事研究 + 民族志研究+ 焦点访谈 + 半结构式访谈)　88

（2）VIP Database　3

#1　（新护士 + 新入职护士 + 新毕业护士 + 新执业护士）

#2　（ICU + 重症监护室 + 重症 + 危重症 + 重症监护 + 监护室）

#3　（规范化培训 + 岗前培训 + 轮转）

#4　（体验 + 感受 + 体会 + 看法 + 经历 + 想法）

#5　（质性研究 + 定性研究 + 现象学 + 扎根理论 + 叙事研究 + 民族志研究+ 焦点访谈 + 半结构式访谈）

#6　#1 AND #2 AND #3 AND #4 AND #5　3

（3）WanFang Data Knowledge Service Platform　19

#1　（新护士 OR 新入职护士 OR 新毕业护士 OR 新执业护士）

#2　（ICU OR 重症监护室 OR 重症 OR 危重症 OR 重症监护 OR 监护室）

#3　（规范化培训 OR 岗前培训 OR 轮转）

#4　（体验 OR 感受 OR 体会 OR 看法 OR 经历 OR 想法）

#5　（质性研究 OR 定性研究 OR 现象学 OR 扎根理论 OR 叙事研究 OR 民族志研究 OR 焦点访谈 OR 半结构式访谈）

#6　#1 AND #2 AND #3 AND #4 AND #5　19

（4）Chinese biological Medical database，CBM　5

检索条件：(((((((("新护士"[常用字段:智能] OR "新入职护士"[常用字段:智能] OR "新毕业护士"[常用字段:智能] OR "新执业护士"[常用字段:智能])) AND (("ICU"[常用字段:智能] OR "重症监护室"[常用字段:智能] OR "监护室"[常用字段:智能] OR "危重症"[常用字段:智能] OR "重症监护"[常用字段:智能] OR "重症"[常用字段:智能])))) AND (("规范化培训"[全部字段:智能] OR "岗前培训"[全部字段:智能] OR "轮转"[全部字段:智能])))) AND (("体验"[常用字段:智能] OR "感受"[常用字段:智能] OR "体会"[常用字段:智能] OR "看法"[常用字段:智能] OR "经历"[常用字段:智能] OR "想法"[常用字段:智能])))) AND (("质性研究"[常用字段:智能] OR "定性研究"[常用字段:智能] OR "现象学"[常用字段:智能] OR "扎根理论"[常用字段:智能] OR "叙事研究"[常用字段:智能] OR "民族志研究"[常用字段:智能] OR "焦点访谈"[常用字段:智能] OR "半结构式访谈"[常用字段:智能])))

（5）PubMed　　10

#1　((((newly graduated nurses[Title/Abstract]) OR (newly qualified nurses[Title/Abstract])) OR (newly employed nurses[Title/Abstract])) OR (newly registered nurses[Title/Abstract])) OR (newly licensed nurses[Title/Abstract])　　730

#2 Intensive Care Units[MeSH Terms] 106485
#3 (((Intensive Care Units[Title/Abstract]) OR (Intensive Care Unit[Title/Abstract])) OR (Unit, Intensive Care[Title/Abstract])) OR (ICU Intensive Care Units[Title/Abstract]) 158192
#4 #2 OR #3 203525
(Intensive Care Units[MeSH Terms]) OR ((((Intensive Care Units[Title/Abstract]) OR (Intensive Care Unit[Title/Abstract])) OR (Unit, Intensive Care[Title/Abstract])) OR (ICU Intensive Care Units[Title/Abstract]))
#5 ((Train[Title/Abstract]) OR (standardized training[Title/Abstract])) OR (pre-service training[Title/Abstract]) 48970
#6 (experience[Title/Abstract]) OR (feel[Title/Abstract]) 869790
#7 qualitative research[MeSH Terms] 834999
#8 ((((((((((((((((((((((qualitative research[Title/Abstract]) OR (qualitativ method[Title/Abstract])) OR (qualitative study[Title/Abstract])) OR (phenomenolog*[Title/Abstract])) OR (lived experience[Title/Abstract])) OR (hermeneutic*[Title/Abstract])) OR (Heideggerian[Title/Abstract])) OR (husserl*[Title/Abstract])) OR (grounded theory[Title/Abstract])) OR (ethnograph*[Title/Abstract])) OR (case study[Title/Abstract])) OR (discourse analy*[Title/Abstract])) OR (interview*[Title/Abstract])) OR (action research[Title/Abstract])) OR (Olparticipant observ*[Title/Abstract])) OR (field note*[Title/Abstract])) OR (focus group*[Title/Abstract])) OR (Colaizzi[Title/Abstract])) OR (content analy*[Title/Abstract])) OR (thematic analy*[Title/Abstract])) OR (Giorgi[Title/Abstract])) OR (Manen[Title/Abstract])) OR (constant comparison[Title/Abstract])) OR (constant comparative analysis[Title/Abstract])
#9 #7 OR #8
(qualitative research[MeSH Terms])OR ((((((((((((((((((((((qualitative research[Title/Abstract]) OR (qualitativ method[Title/Abstract])) OR (qualitative study[Title/Abstract])) OR (phenomenolog*[Title/Abstract])) OR (lived experience[Title/Abstract])) OR (hermeneutic*[Title/Abstract])) OR (Heideggerian[Title/Abstract])) OR (husserl[Title/Abstract])) OR (grounded theory[Title/Abstract])) OR (ethnograph[Title/Abstract])) OR (case study[Title/Abstract])) OR (discourse analy[Title/Abstract])) OR (interview[Title/Abstract])) OR (action research[Title/Abstract])) OR (Olparticipant observ[Title/Abstract])) OR (field note[Title/Abstract])) OR (focus group[Title/Abstract])) OR (Colaizzi[Title/Abstract])) OR (content analy[Title/Abstract])) OR (thematic analy[Title/Abstract])) OR (Giorgi[Title/Abstract])) OR (Manen[Title/Abstract])) OR (constant comparison[Title/Abstract])) OR (constant comparative analysis[Title/Abstract]))
#10 #1 AND #4 AND #5 AND #6 AND #9 10
(((((newly graduated nurses[Title/Abstract]) OR (newly qualified nurses[Title/Abstract])) OR (newly employed nurses[Title/Abstract])) OR (newly registered nurses[Title/Abstract])) OR (newly licensed nurses[Title/Abstract])) AND ((Intensive Care Units[MeSH Terms]) OR ((((Intensive Care Units[Title/Abstract]) OR (Intensive Care Unit[Title/Abstract])) OR (Unit, Intensive Care[Title/Abstract])) OR (ICU Intensive Care Units[Title/Abstract]))) AND (((Train[Title/Abstract]) OR (standardized training[Title/Abstract])) OR (pre-service

training[Title/Abstract])) AND ((experience[Title/Abstract]) OR (feel[Title/Abstract])) AND ((qualitative research[MeSH Terms])OR ((((((((((((((((((((((qualitative research[Title/Abstract]) OR (qualitativ method[Title/Abstract])) OR (qualitative study[Title/Abstract])) OR (phenomenolog[Title/Abstract])) OR (lived experience[Title/Abstract])) OR (hermeneutic[Title/Abstract])) OR (Heideggerian[Title/Abstract])) OR (husserl[Title/Abstract])) OR (grounded theory[Title/Abstract])) OR (ethnograph[Title/Abstract])) OR (case study[Title/Abstract])) OR (discourse analy*[Title/Abstract])) OR (interview[Title/Abstract])) OR (action research[Title/Abstract])) OR (Olparticipant observ*[Title/Abstract])) OR (field note*[Title/Abstract])) OR (focus group*[Title/Abstract])) OR (Colaizzi[Title/Abstract])) OR (content analy*[Title/Abstract])) OR (thematic analy*[Title/Abstract])) OR (Giorgi[Title/Abstract])) OR (Manen[Title/Abstract])) OR (constant comparison[Title/Abstract])) OR (constant comparative analysis[Title/Abstract])))

（6）Web of Science 4

#1 ((((TS=(newly graduated nurses)) OR TS=(newly qualified nurses)) OR TS=(newly employed nurses)) OR TS=(newly registered nurses)) OR TS=(newly licensed nurses) 1594

#2 (((TS=(Intensive Care Unit)) OR TS=(ICU)) OR TS=(Unit,Intensive Care)) OR TS=(ICU Intensive Care Units) 218153

#3 ((TS=(Train)) OR TS=(standardized training)) OR TS=(pre-service training)

#4 (TS=(feel)) OR TS=(experience)

#5 (((((((((((((((((((((TS=(qualitative research)) OR TS=(qualitativ method)) OR TS=(qualitative study)) OR TS=(phenomenolog)) OR TS=(lived experience)) OR TS=(hermeneutic)) OR TS=(Heideggerian)) OR TS=(husserl)) OR TS=(grounded theory)) OR TS=(ethnograph)) OR TS=(case study)) OR TS=(discourse analy)) OR TS=(interview)) OR TS=(action research)) OR TS=(Olparticipant observe)) OR TS=(field note)) OR TS=(focus group)) OR TS=(Colaizzi)) OR TS=(content analy)) OR TS=(thematic analy)) OR TS=(Giorgi)) OR TS=(Manen)) OR TS=(constant comparison)) OR TS=(constant comparative analysis)

#6 1# AND #2 AND 3#AND 4# AND 5# 4

(((((TS=(newly graduated nurses)) OR TS=(newly qualified nurses)) OR TS=(newly employed nurses)) OR TS=(newly registered nurses)) OR TS=(newly licensed nurses)) AND ((((TS=(Intensive Care Unit)) OR TS=(ICU)) OR TS=(Unit,Intensive Care)) OR TS=(ICU Intensive Care Units)) AND (((TS=(Train)) OR TS=(standardized training)) OR TS=(pre-service training)) AND ((TS=(feel)) OR TS=(experience)) AND ((((((((((((((((((((((TS=(qualitative research)) OR TS=(qualitativ method)) OR TS=(qualitative study)) OR TS=(phenomenolog)) OR TS=(lived experience)) OR TS=(hermeneutic)) OR TS=(Heideggerian)) OR TS=(husserl)) OR TS=(grounded theory)) OR TS=(ethnograph)) OR TS=(case study)) OR TS=(discourse analy)) OR TS=(interview)) OR TS=(action research)) OR TS=(Olparticipant observe)) OR TS=(field note)) OR TS=(focus group)) OR TS=(Colaizzi)) OR TS=(content analy)) OR TS=(thematic analy)) OR TS=(Giorgi)) OR TS=(Manen)) OR TS=(constant comparison)) OR TS=(constant comparative analysis))

（7）The Cochrane Library 1

#1 (newly graduated nurses)Mesh 20

#2 (newly graduated nurses):ti,ab,kw OR (newly qualied nurses):ti,ab,kw OR (newly employed nurses)ti,ab,kw oR (newy registered nurses):,ab,kw oR (newy licensed nurses):ti.ab.kw 180

#3 (intensive Care Unit);ti,ab,kw OR (icu):ti,ab,kw OR (Unit.lintensive Care):ti,ab,kw OR (lcu intensive Care Units):ti,ab,kw 31393

#4 (Train):ti,ab,kw OR (standardized training):ti,ab,kw OR (pre-service training):ti,ab,kw 11535

#5 (experience):ti,ab,kw OR (feel):ti,ab,kw 70552

#6 (qualtative research):ti,ab,kw OR (qualltativ method):ti,ab,kw OR (qualtative study):ti,ab,kw OR (phenomenolog):ti,ab,kw OR (lived experience):ti,ab,kw 19935

#7 (hermeneutic):ti,ab,kw OR (Heideggerian):ti,ab,kw OR (husserl):t,ab,kw OR (grounded theory):ti,ab,kw OR (ethnograph):ti,ab,kw 764

#8 (case studyrtlab.kw OR idiscourse analvrilab,kw oR linteniewiyi ab,kw OR lacion research:ti,ab,kw OR l0lparicipant observer':ti,ab,kw 97119

#9 (tfield note):ti,ab,kw OR (focus group):ti,ab,kw OR (Colaizzi):ti,ab,kw OR (content analy):ti,ab,kw OR (thematic analy):ti,ab,kw 17609

#10 (Manen):ti,ab,kw OR (constant comparison)::ti,ab,kw OR (constant comparative analysis)::ti,ab,kw 2448

#11 #6 OR #7OR #8 OR #9 OR #10

#12 #2 AND #3 AND #4 1

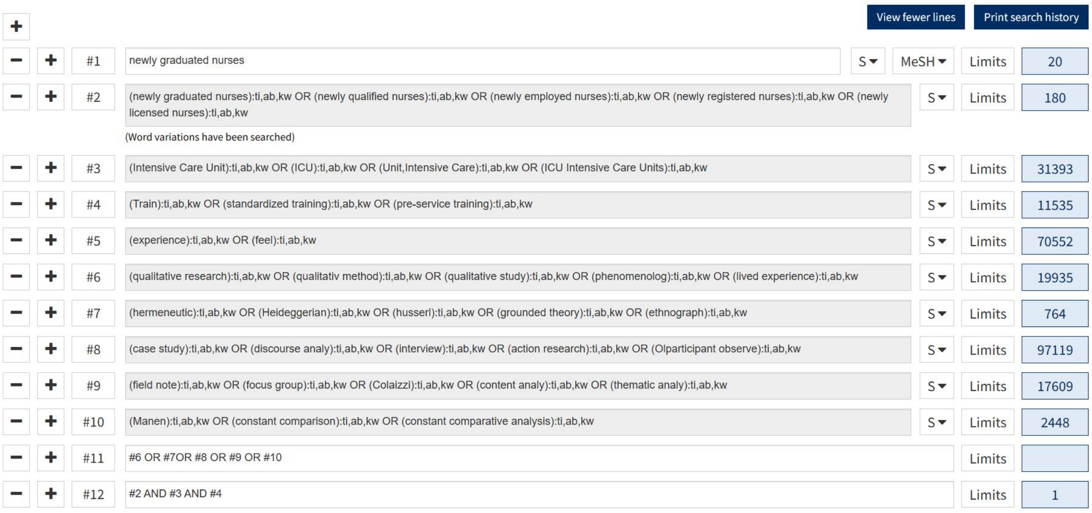

FIGURE 1

（8）Embase 1

#1 'newly graduated nurses' OR (newly AND graduated AND ('nurses'exp OR nurses) OR 'newly qualified nurses':ab,ti OR 'newly employed nurses':ab,ti OR 'newly registered nurses':ab.ti OR 'newly licensed nurses':ab ti 850

#2 'intensive care unit'/exp OR "intensive care unit OR (intensive AND ('care'exp OR care) AND ('unit/exp OR unit) OR icu:ab.ti OR 'unit,intensive care':ab.ti OR 'icu intensive care units':ab.ti 494914

#3 'train'/exp OR train OR 'standardized training':ab,ti OR 'pre-service training':ab,ti

#4 'experience'/exp OR experience OR feel:ab,ti 1338358

#5 'qualitative research'exp OR 'qualitative research' OR (('qualitative'exp OR qualitative) AND ('research'exp OR research) OR qualitative study':ab,ti OR phenomenology:ab,ti OR 'qualitative method':ab,ti 294423

#6 #1 AND #2 AND #3 AND #4 1

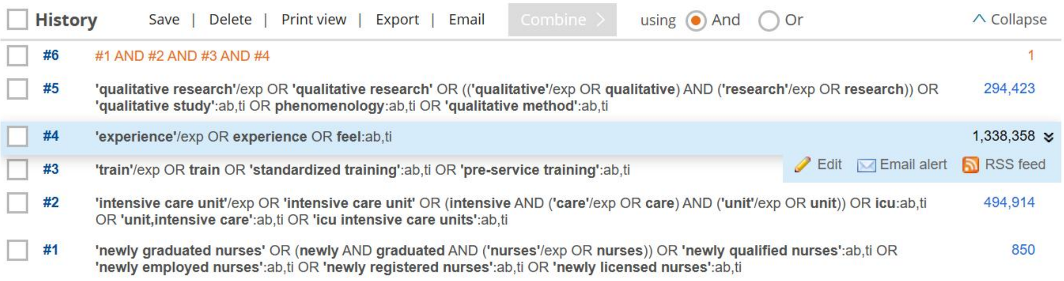

FIGURE 2

（9）CINAHL (EBSCO) 45

#1 newly graduated nurses OR newly qualified nurses OR newly employed nurses OR newly registered nurses OR newly licensed nurses 3773

#2 Intensive Care Unit OR ICU OR Unit,Intensive Care OR ICU Intensive Care Units 535232

#3 train OR standardized training OR pre-service training 760214

#4 experience OR fee 6273030

#5 Qualitative research OR qualitative study OR phenomenology OR Qualitative method

#6 #1 AND #2 AND #3 AND #4 AND #5 45

NIHR | National Institute for Health and Care Research

PROSPERO
International prospective register of systematic reviews

Home | About PROSPERO | How to register | Service information   Search | My PROSPERO | Logout: **ligang wang**

Register your review now   Edit your details

You have 2 records

My other records

These are records that have either been published or rejected and are not currently being worked on.

ID	Title	Status	Last edited
CRD42023475257	The experiences of Newly Qualified Nurses in Intensive Care Unit: A qualitative meta- synthesis<br>To enable PROSPERO to focus on COVID-19 registrations during the 2020 pandemic, this registration record was automatically published exactly as submitted. The PROSPERO team has not checked eligibility.	Registered	02/11/2023
